# Association between oxidative balance score and glaucoma in the National Health and Nutrition Examination Survey

**DOI:** 10.3389/fnut.2025.1528114

**Published:** 2025-01-15

**Authors:** Jun Huang, Yulan Zhang, Chao Wu, Yifan Wu, Feiran Wang, Yunxuan Ning, Lu Shi

**Affiliations:** Department of Ophthalmology, The Second Affiliated Hospital, Nanchang University, Nanchang, China

**Keywords:** oxidative balance score, glaucoma, oxidative stress, National Health and Nutrition Examination Survey (NHANES), comprehensive index

## Abstract

**Objective:**

To investigate the association between Oxidative Balance Score (OBS) and glaucoma risk.

**Methods:**

Using data from the National Health and Nutrition Examination Survey (2005–2008), we analyzed 2,615 participants aged ≥40 years. OBS was calculated from 15 antioxidant and 5 pro-oxidant components, including dietary nutrients and lifestyle factors. Glaucoma was identified through self-reported diagnosis and retinal imaging. Survey-weighted logistic regression and restricted cubic splines (RCS) were used to assess associations.

**Results:**

Higher OBS was associated with lower glaucoma risk (adjusted OR = 0.97, 95% CI: 0.94–1.00). Participants in the highest OBS quartile showed 51% lower odds of glaucoma compared to the lowest quartile (adjusted OR = 0.49, 95% CI: 0.27–0.90). The protective effect was more pronounced among middle-aged adults (40–60 years; OR = 0.90, 95% CI: 0.86–0.95) and males (OR = 0.93, 95% CI: 0.90–0.97).

**Conclusion:**

Higher OBS were associated with lower glaucoma prevalence, particularly among middle-aged adults and males, suggesting potential benefits of maintaining oxidative balance in glaucoma prevention.

## Introduction

Glaucoma, a progressive optic neuropathy, is one of the leading causes of irreversible blindness worldwide. According to the latest Global Burden of Disease study, approximately 76.1 million people were affected by glaucoma globally in 2020, with projections indicating an increase to 95.1 million by 2030 (1). In China, the prevalence of glaucoma is about 2.58% and increases with age (2). This disease not only significantly impacts patients’ quality of life but also poses a substantial burden on society and healthcare systems.

Oxidative stress plays a crucial role in the pathogenesis of glaucoma, primarily through the production of reactive oxygen species (ROS) leading to optic nerve damage and retinal ganglion cell death (3). Antioxidants play a vital role in modulating ocular oxidative stress, potentially offering new strategies for glaucoma prevention and treatment (4). Among various antioxidants, vitamins C and E demonstrate particular efficacy in mitigating oxidative stress-induced damage to retinal ganglion cells, with clinical studies revealing decreased serum vitamin C levels in patients with normal-tension glaucoma (5). Beyond direct antioxidant effects, dietary fiber indirectly modulates immune status and oxidative stress levels through improvements in gut microbiota composition (6, 7). The role of trace elements warrants particular attention. Copper, generally considered to have antioxidant properties in human metabolism, has been found at elevated concentrations in both the serum and aqueous humor of glaucoma patients (8). Furthermore, magnesium deficiency acts as a pathogenic factor that increases oxidative stress and induces nitric oxide synthase (NOS) activity, potentially contributing to the onset and progression of glaucoma (9). Lifestyle factors significantly influence oxidative stress levels, with smoking and excessive alcohol consumption serving as major contributors (10). Moreover, an epidemiological study has demonstrated that a lower body mass index (BMI) (<19 kg/m^2^) is associated with an increased risk of open-angle glaucoma (11). However, the complex interactions and potential synergistic effects among these various factors highlight the limitations of single-factor studies in accurately assessing overall oxidative stress status, necessitating a more comprehensive and integrated evaluation approach.

The Oxidative Balance Score (OBS) is a comprehensive index that assesses an individual’s exposure to antioxidants and pro-oxidants through diet and lifestyle (12). By integrating multiple factors, including dietary intake and lifestyle habits, OBS provides a holistic evaluation of oxidative stress (13). A higher OBS indicates greater exposure to antioxidants and lower exposure to pro-oxidants, thereby reflecting a more favorable oxidative balance status with potentially lower levels of oxidative stress. In recent years, numerous studies have demonstrated strong correlations between OBS and the risk and prognosis of various chronic diseases. For instance, higher OBS has been significantly associated with reduced cardiovascular disease risk (14), improved metabolic syndrome (15), and decreased all-cause mortality (16). These findings highlight the potential value of OBS in assessing health status and predicting disease risk.

However, despite evidence linking oxidative stress to glaucoma, there is currently a lack of large-scale population studies directly exploring the specific relationship between OBS and glaucoma. This research gap limits our understanding of the role of dietary and lifestyle factors in glaucoma development.

This paper aims to investigate the association between OBS and the risk of glaucoma based on a cross-sectional study of data from the National Health and Nutrition Examination Survey (NHANES) in the United States, with the hope of providing a new perspective for the prevention and early intervention of glaucoma.

## Materials and methods

### Sample and population

This study utilized data from two NHANES survey cycles (2005–2006 and 2007–2008), which provided the most comprehensive ophthalmological assessments. It was approved by the Research Ethics Committee of the National Center for Health Statistics (NCHS). As this was a retrospective analysis using publicly available data with anonymized participant information, individual informed consent was not required. The study adhered to the ethical principles of the Declaration of Helsinki, ensuring participant privacy and data security. All research procedures complied with Institutional Review Board requirements and international research ethics standards. Strict data protection measures were implemented to maintain participant confidentiality and information security throughout the study process.

**Figure 1:**
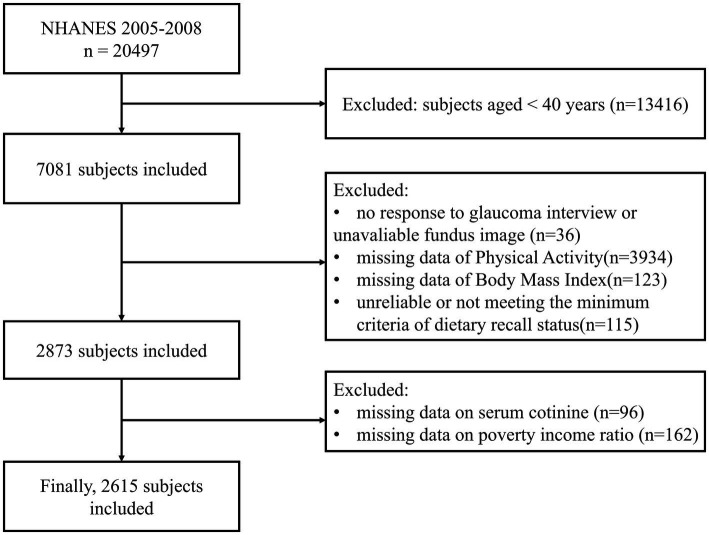
Flowchart for participants recruitment of this study, NHANES 2005–2008.

The study population includes NHANES participants aged 40 years and older. A multistage stratified sampling method was used, initially including 7,081 participants. Exclusion criteria were: non-response to the glaucoma questionnaire or unreliable Retinal Imaging (*n* = 36), lack of physical activity data (*n* = 3,934), missing Body Mass Index (BMI) data (*n* = 123), unreliable or not meeting the minimum criteria of dietary recall status (*n* = 115), missing serum cotinine data (*n* = 96), and missing poverty income ratio (PIR) data (*n* = 162). Ultimately, 2,615 eligible participants were included in the present study (Figure 1).

### Exposure variable

The Oxidative Balance Score (OBS) comprises 15 antioxidant and 5 pro-oxidant components, including 16 nutrients (dietary fiber, carotene, riboflavin, niacin, vitamin B6, total folate, vitamin B12, vitamin C, vitamin E, calcium, iron, magnesium, zinc, copper, selenium and total fat) and 4 lifestyle factors (BMI, cotinine, alcohol and physical activity). Data on the 16 nutrients and alcohol consumption were obtained from the NHANES dietary interview data, with the carotene value representing the sum of Alpha-carotene and Beta-carotene. The first dietary recall was collected in person during the NHANES visit, and the second was collected by telephone 3 to 10 days later. The mean of the two days’ values was used for our analysis. If the dietary data for the second day was missing, the first day’s data was used instead. Dietary supplements were not included in our study. Serum cotinine, the primary metabolite of nicotine, was used to estimate exposure to environmental tobacco smoke and thus reflected smoking status. BMI was obtained from the examination data. Physical activity data were sourced from household interviews; we included data on leisure-time physical activity over the past 30 days to calculate weekly metabolic equivalents (MET). MET scores = weekly frequency of each physical activity * duration of each physical activity* each physical activity suggested MET Scores. For alcohol consumption, individuals were classified as non-drinkers, non-heavy drinkers (0 to 30 g/d for male, 0 to 15 g/d for female), and heavy drinkers (≥30 g/d for male, ≥15 g/d for female), with scores of 2, 1, and 0 points assigned, respectively. Other components were categorized into three groups according to their gender-specific tertile distributions. For antioxidant components, scores of 0, 1, and 2 were assigned to the first, second, and third tertiles, respectively. For pro-oxidant components, the scoring was reversed, with 0 points assigned to the highest tertile and 2 points to the lowest tertile. Based on the algorithm by Zhang et al. (17), the OBS score was derived from the sum of scores assigned to all components, with the total score ranging from 0 to 40 points. The detailed oxidative balance score assignment scheme is shown in Table 1.

**Table 1:** Oxidative balance score assignment scheme.

Components	Property		Male			Female	
		0	1	2	0	1	2
Dietary fiber (gm/d)	A	<14.2	14.2–21.3	≥21.3	<12	12–17.5	≥17.5
Carotene (mcg/d)	A	<922	922–2,998	≥2,998	<926	926–3,291	≥3,291
Riboflavin (mg/d)	A	<1.94	1.94–2.72	≥2.72	<1.49	1.49–2.09	≥2.09
Niacin (mg/d)	A	<22.1	22.1–31.5	≥31.5	<16.1	16.1–23.0	≥23.0
Vitamin B6 (mg/d)	A	<1.76	1.76–2.57	≥2.57	<1.31	1.31–1.90	≥1.90
Total folate (mcg/d)	A	<346	346–516	≥516	<269	269–394	≥394
Vitamin B12 (mcg/d)	A	<3.90	3.90–6.60	≥6.60	<2.80	2.80–4.80	≥4.80
Vitamin C (mg/d)	A	<53.5	53.5–117	≥117	<49.6	49.6–100	≥100
Vitamin E (mg/d)	A	<5.83	5.83–8.91	≥8.91	<4.78	4.78–7.52	≥7.52
Calcium (mg/d)	A	<732	732–1,081	≥1,081	<618	618–890	≥890
Iron (mg/d)	P	≥19.8	13.6–19.8	<13.6	≥14.8	10.3–14.8	<10.3
Magnesium (mg/d)	A	<268	268–370	≥370	<218	218–302	≥302
Zinc (mg/d)	A	<10.2	10.2–14.7	≥14.7	<7.5	7.5–10.8	≥10.8
Copper (mg/d)	A	<1.17	1.17–1.62	≥1.62	<0.95	0.95–1.32	≥1.32
Selenium (mcg/d)	A	<97.4	97.4–137	≥137	<71.4	71.4–101	≥101
Total Fat (g/d)	P	≥98.2	65.1–98.2	<65.1	≥71.5	48.8–71.5	<48.8
BMI (kg/m^2^)	P	≥29.9	26.3–29.9	<26.3	≥30.5	25.2–30.5	<25.2
Cotinine (ng/mL)	P	≥0.241	0.025–0.241	<0.025	≥0.064	0.018–0.064	<0.018
Physical Activity (MET-minute/week)	A	<569	569–1,575	≥1,575	<420	420–1,129	≥1,129
Alcohol (g/d)	P	≥30	0–30	None	≥15	0–15	None

OBS, oxidative balance score; A, antioxidant; P, prooxidant; BMI, body mass index; MET, metabolic equivalent.

### Outcome variable

Outcome variables were collected through vision questionnaires and examination data (retinal imaging). The vision questionnaire was administered to participants aged 40 years and older. Participants who responded “Do not know,” refused to answer or had missing data for the question “Have you ever been told by an eye doctor that you have glaucoma, sometimes called high pressure in your eyes?” were excluded from the analysis. Retinal imaging examinations were conducted using ophthalmic digital imaging systems (Retinal Photography). All examination participants were excluded if they were blindness, had eye infections, or wore eye patches over both eyes. In 2012, ophthalmologists from Johns Hopkins University re-evaluated retinal images of participants aged 40 years and older with a vertical cup-to-disc ratio (CDR) of 0.6 or greater to identify characteristics suggestive of glaucoma. The likelihood of glaucoma was categorized into five grades (No, Possible, Probable, Definite, Ungradable). Participants were considered to have glaucoma if either eye received a consensus expert grade of “Probable” or “Definite.”

### Covariates

Multiple potential confounding factors were included as covariates in the analysis. Covariates included age, gender, race/ethnicity (categorized as Mexican American, Other Hispanic, Non-Hispanic White, Non-Hispanic Black, Other Race), education level (categorized as Less than High School, High School Graduate, College or Above), marital status (Never Married, Married/Living with partner, Divorced/Separated/Widowed), poverty income ratio (PIR), hypertension, high cholesterol, diabetes, and kidney disease. PIR was graded into three categories based on the analysis guideline: ≤1.3, 1.3–3.5, and > 3.5 (18). Information regarding hypertension, high cholesterol, diabetes, and kidney disease was obtained from self-reported questionnaire data.

### Statistical analysis

All statistical analyses were performed using R software (version 4.4.0). All analyses incorporated sampling weights (WTDRD1) and complex survey design features of NHANES to ensure nationally representative estimates. Baseline characteristics were presented as survey-weighted means with 95% confidence intervals (CIs) for continuous variables and survey-weighted percentages with 95% CIs for categorical variables. Survey-weighted linear regression for continuous variables and survey-weighted Chi-square tests for categorical variables were used to examine differences across OBS quartiles. The association between OBS and glaucoma was evaluated using survey-weighted multiple logistic regression models, with progressive adjustment for potential confounders: Model 1 (unadjusted), Model 2 (adjusted for age and gender), Model 3 (further adjusted for race/ethnicity, education level, marital status, and PIR), and Model 4 (additionally adjusted for hypertension, high cholesterol, diabetes, and kidney disease). To assess the dose–response relationship, OBS was analyzed both as a continuous variable and as quartiles, with P-trend calculated across quartiles. Restricted cubic spline analysis was performed to examine potential non-linear relationships between OBS and glaucoma probability. Subgroup analyses were conducted to evaluate effect modification by age, gender, PIR, diabetes and hypertension, with interaction terms tested using Wald tests. All statistical tests were two-sided, with *p* < 0.05 considered statistically significant.

## Result

### Demographic characteristics of the study sample

Table 2 presents the baseline characteristics across oxidative balance score (OBS) quartiles. Age and gender distributions were comparable across quartiles (*p* = 0.0702 and *p* = 0.6198, respectively). Significant sociodemographic differences were observed, with higher OBS quartiles showing increased proportions of Non-Hispanic Whites (71.44% in Q1 to 87.04% in Q4, *p* < 0.0001), college graduates (51.95 to 74.37%, *p* < 0.0001), and higher income individuals (PIR > 3.5: 47.26 to 67.47%, *p* < 0.0001).

**Table 2:** Baseline characteristics of participants by quartiles of the OBS.

Characteristics	Q1(N = 13,068,760)	Q2(N = 15,120,504)	Q3(N = 17,728,379)	Q4(N = 19,714,723)	*p*-value
Age, years	56.31 (55.13, 57.48)	56.72 (55.48, 57.95)	55.18 (53.85, 56.51)	54.74 (53.21, 56.28)	0.0702
Gender					0.6198
Male	47.82 (42.81, 52.87)	46.72 (41.76, 51.73)	50.96 (45.10, 56.80)	47.23 (42.64, 51.87)	
Female	52.18 (47.13, 57.19)	53.28 (48.27, 58.24)	49.04 (43.20, 54.90)	52.77 (48.13, 57.36)	
Race/ethnicity					<0.0001
Mexican American	4.95 (3.60, 6.77)	4.63 (3.37, 6.33)	4.23 (2.92, 6.11)	2.59 (1.75, 3.80)	
Other Hispanic	3.67 (2.17, 6.14)	1.53 (0.77, 3.04)	1.81 (0.99, 3.28)	1.72 (0.98, 3.01)	
Non-Hispanic White	71.44 (66.15, 76.20)	79.62 (73.25, 84.79)	82.56 (78.34, 86.10)	87.04 (82.44, 90.57)	
Non-Hispanic Black	14.94 (10.73, 20.44)	9.17 (6.17, 13.41)	6.99 (5.09, 9.51)	4.11 (2.80, 6.00)	
Other race	4.99 (2.84, 8.62)	5.05 (3.17, 7.94)	4.41 (2.95, 6.54)	4.54 (2.43, 8.30)	
Education level					<0.0001
Less than high school	18.27 (15.25, 21.73)	11.17 (8.89, 13.95)	8.44 (5.24, 13.32)	7.48 (5.14, 10.78)	
High school graduate	29.78 (25.44, 34.51)	23.23 (18.96, 28.14)	23.10 (18.68, 28.20)	18.14 (13.20, 24.41)	
College or above	51.95 (47.30, 56.57)	65.60 (60.41, 70.44)	68.46 (61.93, 74.34)	74.37 (67.62, 80.14)	
PIR					<0.0001
≤1.3	15.76 (13.28, 18.61)	11.82 (9.51, 14.62)	8.80 (6.11, 12.51)	7.66 (4.92, 11.73)	
1.3–3.5	36.98 (31.73, 42.56)	32.81 (27.42, 38.70)	28.38 (22.68, 34.86)	24.87 (20.22, 30.19)	
>3.5	47.26 (41.47, 53.12)	55.36 (48.80, 61.75)	62.82 (54.73, 70.26)	67.47 (61.14, 73.22)	
Marital status					0.0197
Married/living with partner	68.21 (63.62, 72.48)	70.77 (65.44, 75.59)	66.92 (60.43, 72.82)	76.79 (71.82, 81.12)	
Divorced/separated/widowed	27.26 (23.17, 31.77)	22.79 (18.46, 27.79)	25.71 (20.13, 32.21)	18.60 (14.92, 22.94)	
Never married	4.53 (2.71, 7.48)	6.44 (4.66, 8.82)	7.38 (4.71, 11.37)	4.61 (2.59, 8.07)	
Hypertension					0.0007
Yes	45.56 (41.44, 49.75)	43.26 (38.57, 48.08)	35.40 (30.42, 40.73)	35.01 (29.79, 40.61)	
No	54.44 (50.25, 58.56)	56.74 (51.92, 61.43)	64.60 (59.27, 69.58)	64.99 (59.39, 70.21)	
High cholesterol					0.8187
Yes	40.17 (34.21, 46.44)	43.86 (38.24, 49.65)	42.49 (37.10, 48.06)	42.27 (37.72, 46.95)	
No	59.83 (53.56, 65.79)	56.14 (50.35, 61.76)	57.51 (51.94, 62.90)	57.73 (53.05, 62.28)	
Diabetes					0.0011
Yes	9.17 (6.83, 12.21)	11.91 (9.41, 14.96)	6.48 (4.62, 9.01)	5.78 (4.10, 8.08)	
No	90.83 (87.79, 93.17)	88.09 (85.04, 90.59)	93.52 (90.99, 95.38)	94.22 (91.92, 95.90)	
Kidney disease					0.0029
Yes	2.29 (1.08, 4.77)	3.02 (1.68, 5.37)	0.63 (0.25, 1.59)	0.73 (0.23, 2.31)	
No	97.71 (95.23, 98.92)	96.98 (94.63, 98.32)	99.37 (98.41, 99.75)	99.27 (97.69, 99.77)	
Glaucoma					0.0093
Yes	7.58 (5.47, 10.42)	6.51 (4.38, 9.58)	3.99 (2.71, 5.83)	3.86 (2.56, 5.79)	
No	92.42 (89.58, 94.53)	93.49 (90.42, 95.62)	96.01 (94.17, 97.29)	96.14 (94.21, 97.44)	

Data are presented as survey-weighted mean (95% CI) for continuous variables and survey-weighted percentage (95% CI) for categorical variables. *p*-values were calculated using survey-weighted linear regression for continuous variables and survey-weighted Chi-square test for categorical variables. *N* represents weighted frequency counts.

OBS, oxidative balance score; PIR, poverty income ratio.

Health conditions showed notable variations across OBS quartiles. The prevalence of hypertension (45.56 to 35.01%, *p* = 0.0007), diabetes (9.17 to 5.78%, *p* = 0.0011), kidney disease (2.29 to 0.73%, *p* = 0.0029), and glaucoma (7.58 to 3.86%, *p* = 0.0093) decreased significantly from Q1 to Q4. High cholesterol prevalence remained consistent across quartiles (*p* = 0.8187). Marital status differed significantly (*p* = 0.0197), with the highest proportion of married/partnered participants in Q4 (76.79, 95% CI: 71.82–81.12%).

### Association between OBS and risk of glaucoma

The association between oxidative balance score (OBS) and glaucoma risk was examined using multiple logistic regression models (Table 3). Analysis of OBS as a continuous variable revealed a significant inverse association with glaucoma risk. In the fully adjusted model accounting for demographic characteristics, socioeconomic factors, and comorbidities, each unit increase in OBS was associated with a 3% reduction in glaucoma risk (OR = 0.97, 95% CI: 0.94–1.00, *p* = 0.044).

**Table 3:** Logistic regression models for glaucoma and OBS.

Models	Continuous OBS		OBS Quartiles, OR (95% CI)				
	OR (95% CI)	*p* value	Q1	Q2	Q3	Q4	P-trend
Model 1	0.96 (0.93, 0.98)	0.001	1.00 (ref)	0.53 (0.32, 0.89) *	0.49 (0.27, 0.89) *	0.40 (0.23, 0.69) **	0.001
Model 2	0.96 (0.94, 0.99)	0.007	1.00 (ref)	0.54 (0.31, 0.96) *	0.54 (0.29, 1.02)	0.47 (0.27, 0.80) **	0.009
Model 3	0.97 (0.94, 1.00)	0.043	1.00 (ref)	0.56 (0.30, 1.05)	0.57 (0.28, 1.15)	0.50 (0.26, 0.94) *	0.039
Model 4	0.97 (0.94, 1.00)	0.044	1.00 (ref)	0.51 (0.27, 0.96) *	0.58 (0.28, 1.18)	0.49 (0.27, 0.90) *	0.041

OR, Odds Ratio; CI, Confidence Interval; OBS, Oxidative Balance Score. *p < 0.05, **p < 0.01.

Model 1: Unadjusted.

Model 2: Adjusted for age and gender.

Model 3: Adjusted for age, gender, race/ethnicity, education level, marital status, and PIR.

Model 4: Adjusted for age, gender, race/ethnicity, education level, marital status, PIR, hypertension, high cholesterol, diabetes, and kidney disease.

Quartile analysis demonstrated a more pronounced protective effect. In the unadjusted model, compared to the lowest OBS quartile, participants in higher quartiles showed substantially lower odds of glaucoma, with risk reductions of 47% in Q2 (OR = 0.53, 95% CI: 0.32–0.89), 51% in Q3 (OR = 0.49, 95% CI: 0.27–0.89), and 60% in Q4 (OR = 0.40, 95% CI: 0.23–0.69), exhibiting a significant dose–response relationship (P-trend = 0.001). Notably, this protective association persisted after comprehensive adjustment for potential confounders (Model 4). In the fully adjusted model, participants in the highest OBS quartile maintained a 51% lower odds of glaucoma compared to the lowest quartile (OR = 0.49, 95% CI: 0.27–0.90), while those in Q2 showed a similar magnitude of protection (OR = 0.51, 95% CI: 0.27–0.96). The significant trend across quartiles (P-trend = 0.041) suggests a robust dose-dependent relationship between higher OBS and lower glaucoma risk, independent of various demographic, socioeconomic, and health-related factors.

**Figure 2:**
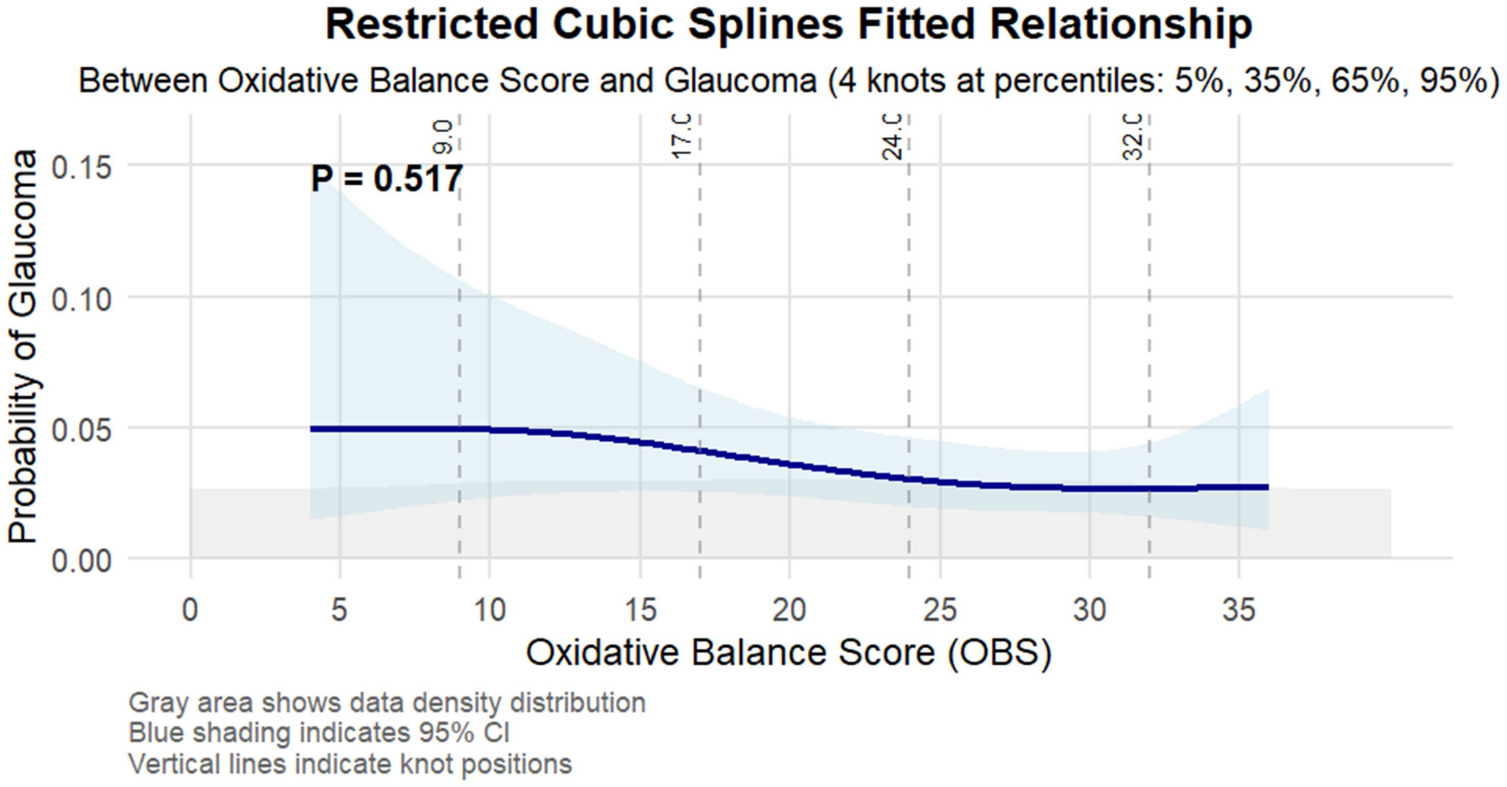
Restricted cubic splines fitted relationship between oxidative balance score and glaucoma. The light blue area represents the 95% confidence interval of the fit. Adjusted for age, gender, race/ethnicity, education level, marital status, PIR, hypertension, high cholesterol, diabetes, and kidney disease.

To further characterize this association, restricted cubic spline analysis (Figure 2) revealed a non-linear inverse relationship between OBS and glaucoma probability, with the strongest association observed in the middle range of OBS values (15–25). The statistical reliability of this relationship was particularly robust in regions with high data density, as indicated by narrow confidence intervals.

### Subgroup analyses

**Figure 3:**
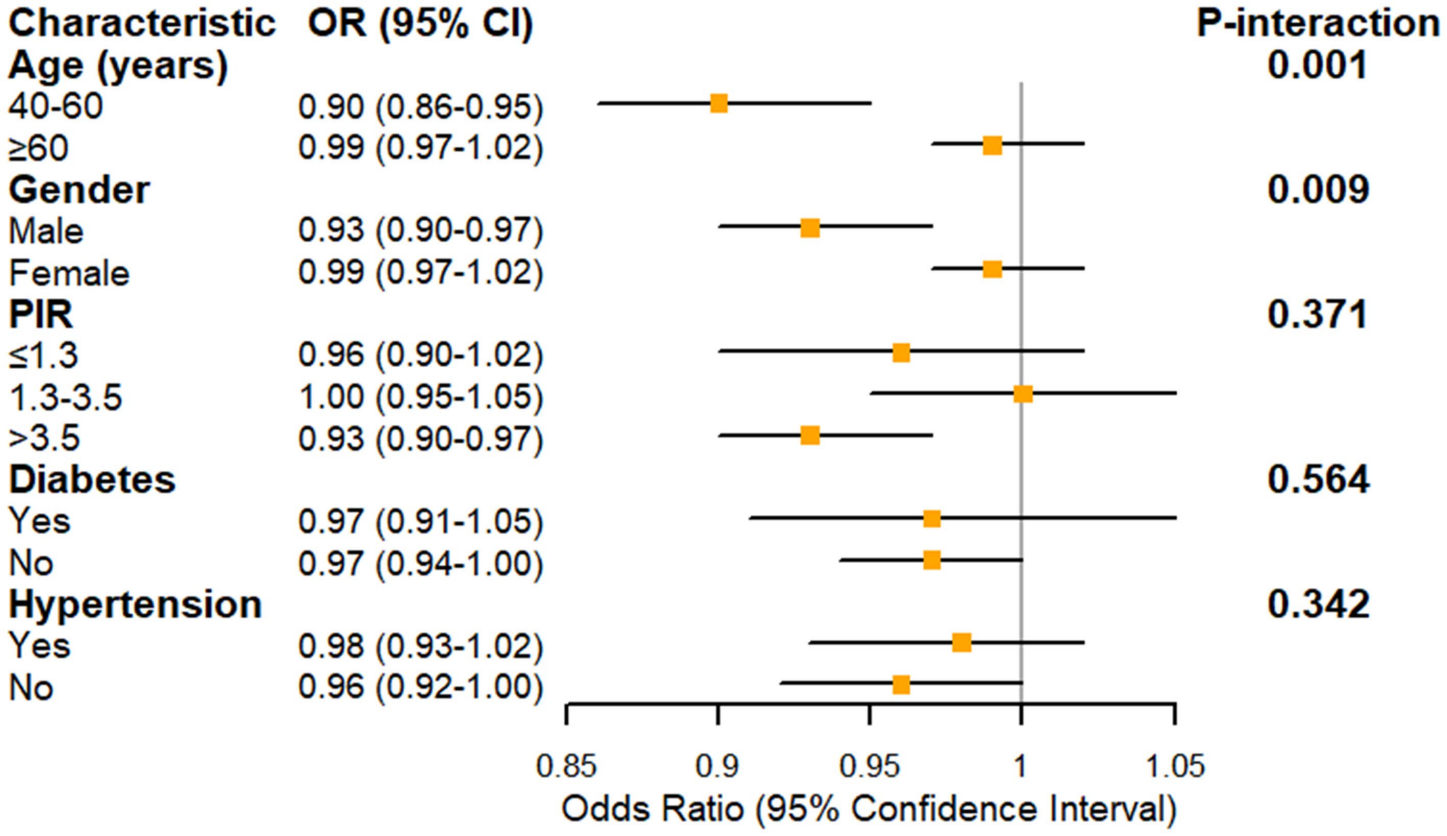
Forest plot showing the association between oxidative balance score and glaucoma prevalence stratified by demographic and clinical characteristics. PIR, poverty income ratio. Model was adjusted for age, gender, race/ethnicity, education level, PIR, marital status, hypertension, cholesterol, diabetes, and kidney disease.

Extending these findings, subgroup analysis revealed significant effect modifications by age and gender in the association between oxidative balance score (OBS) and glaucoma prevalence (Figure 3). The protective effect of OBS was more pronounced among middle-aged adults (40–60 years) with an odds ratio (OR) of 0.90 (95% CI: 0.86–0.95) compared to older adults (≥60 years; OR = 0.99, 95% CI: 0.97–1.02; P-interaction = 0.001). Similarly, males exhibited a stronger protective association (OR = 0.93, 95% CI: 0.90–0.97) than females (OR = 0.99, 95% CI: 0.97–1.02; P-interaction = 0.009). Among socioeconomic strata, participants with poverty income ratio > 3.5 demonstrated the most robust protective association (OR = 0.93, 95% CI: 0.90–0.97), although the interaction was not statistically significant (P-interaction = 0.371). The protective effect of OBS remained consistent across comorbidity status, with no significant effect modification by diabetes (P-interaction = 0.564) or hypertension (P-interaction = 0.342).

Further quartile-based stratification reinforced these patterns, revealing particularly strong protective effects in specific subgroups (Table 4). Among middle-aged adults (40–60 years), a strong dose–response relationship was evident, with the highest OBS quartile showing a 94% reduced odds of glaucoma (OR = 0.06, 95% CI: 0.01–0.50) compared to the lowest quartile. This protective association was less pronounced in older adults (≥60 years), with no significant differences across OBS quartiles.

**Table 4:** Stratified analysis of the association between OBS quartiles and glaucoma.

Subgroups	OBS Quartiles, OR (95% CI)				P for interaction
	Q1	Q2	Q3	Q4	
Age (years)					0.024
40–60	1.00	0.45 (0.14–1.48)	0.23 (0.06–0.94) *	0.06 (0.01–0.50)*	
≥60	1.00	0.55 (0.28–1.08)	0.85 (0.42–1.69)	0.88 (0.53–1.46)	
Gender					0.025
Male	1.00	0.32 (0.13–0.74)*	0.27 (0.10–0.70)*	0.32 (0.15–0.69)**	
Female	1.00	0.87 (0.45–1.66)	1.35 (0.58–3.12)	0.87 (0.41–1.84)	
PIR					0.094
≤1.3	1.00	0.70 (0.26–1.84)	0.28 (0.11–0.70)*	0.80 (0.24–2.71)	
1.3–3.5	1.00	0.71 (0.33–1.52)	0.82 (0.31–2.17)	1.38 (0.56–3.39)	
>3.5	1.00	0.29 (0.12–0.68)**	0.42 (0.15–1.18)	0.22 (0.10–0.50)**	
Diabetes					0.740
Yes	1.00	0.56 (0.17–1.86)	0.53 (0.13–2.21)	0.61 (0.10–3.68)	
No	1.00	0.51 (0.28–0.92)*	0.58 (0.27–1.24)	0.50 (0.26–0.97)*	
Hypertension					0.088
Yes	1.00	0.28 (0.09–0.84)*	0.63 (0.22–1.77)	0.74 (0.38–1.45)	
No	1.00	0.83 (0.40–1.74)	0.58 (0.21–1.64)	0.32 (0.11–0.94)*	

OR, Odds Ratio; CI, Confidence Interval; OBS, Oxidative Balance Score; PIR, poverty income ratio. *p < 0.05; **p < 0.01; Model was adjusted for age, gender, race/ethnicity, education level, PIR, marital status, hypertension, cholesterol, diabetes, and kidney disease, except for the stratification variable itself. P for interaction was derived from Wald test of the interaction terms between OBS quartiles and the stratification variable.

Gender-stratified analysis revealed a consistent protective effect among males, with similar risk reductions across higher OBS quartiles (ORs ranging from 0.27 to 0.32, all *p* < 0.05). In contrast, females showed no significant associations across OBS quartiles. Socioeconomic stratification suggested a marginally significant interaction (P-interaction = 0.094), with the strongest protective effects observed in the highest socioeconomic group (PIR > 3.5), where the highest OBS quartile was associated with a 78% reduced odds of glaucoma (OR = 0.22, 95% CI: 0.10–0.50).

While diabetes status did not modify the OBS-glaucoma association (P-interaction = 0.740), hypertension showed a marginally significant interaction (P-interaction = 0.088). Among hypertensive individuals, the protective effect was most pronounced in the second OBS quartile (OR = 0.28, 95% CI: 0.09–0.84), while in non-hypertensive individuals, the strongest protection was observed in the highest quartile (OR = 0.32, 95% CI: 0.11–0.94).

## Discussion

This study investigates the relationship between the Oxidative Balance Score (OBS) and the risk of glaucoma. Based on cross-sectional data from the National Health and Nutrition Examination Survey (NHANES) from 2005 to 2008, this research involves a large sample of 2,615 participants, providing a robust foundation for analysis. The results indicate a significant inverse association between OBS and glaucoma prevalence, with a more pronounced protective effect observed among middle-aged adults (40–60 years) (OR = 0.90, 95% CI: 0.86–0.95), while no significant difference was found in older adults (≥60 years) (OR = 0.99, 95% CI: 0.97–1.02). Additionally, the protective effect was stronger in males (OR = 0.93, 95% CI: 0.90–0.97) compared to females, who showed no significant difference (OR = 0.99, 95% CI: 0.97–1.02). These findings offer new perspectives for the prevention and early intervention of glaucoma. While our data is from 2005–2008, the value of this research remains significant. The fundamental biological mechanisms linking oxidative stress to glaucoma development remain consistent over time, and these NHANES cycles uniquely provided comprehensive ophthalmological assessments with expert validation from Johns Hopkins University. Our findings serve as an important baseline reference for understanding the relationship between oxidative balance and glaucoma, contributing to the identification of modifiable risk factors for glaucoma prevention that remains relevant for current clinical practice.

Oxidative stress has been identified as a critical pathogenic mechanism in glaucoma. Tezel et al. (24) demonstrated that oxidative stress leads to mitochondrial dysfunction and DNA damage in retinal ganglion cells (RGCs), subsequently triggering apoptotic pathways. These cellular alterations ultimately manifest as optic nerve atrophy and irreversible vision loss. In a comprehensive systematic review and meta-analysis, Benoist d’Azy et al. (19) reported significantly elevated levels of oxidative stress markers coupled with markedly reduced antioxidant levels in glaucoma patients, providing compelling evidence for the fundamental role of oxidative stress in glaucomatous pathogenesis.

Previous studies have provided substantial evidence regarding the relationship between individual components of OBS and glaucoma. Liu et al. (21) identified a significant association between high-dose vitamin B12 intake and glaucoma occurrence. Additionally, a NHANES-based study (23) revealed that higher niacin intake might be associated with reduced odds of glaucoma. Furthermore, adequate dietary intake of Ca, K, and Mg has been suggested as potential protective factors against glaucoma (25). In a noteworthy investigation, Ramdas et al. (22) demonstrated that intake of *β*-carotene, retinol, vitamin B1, and vitamin B12 was inversely associated with the risk of open-angle glaucoma. Consistent with these findings, Li et al. (20) reported that each unit increase in dietary antioxidant index corresponded to a 6% reduction in self-reported glaucoma risk [0.94 (0.90, 0.99), *p* = 0.02]. Conversely, Wang et al. (26) observed that increased total iron intake might be associated with higher odds of glaucoma. Of particular interest, Yang et al. (27) found that individuals in the highest quartile of vitamin B6 intake exhibited a 75% lower risk of glaucoma compared to those in the lowest quartile (OR = 0.25, 95% CI 0.07–0.92). Moreover, in a study of 584 African American women, Giaconi et al. (28) demonstrated that consumption of fruits and vegetables rich in vitamins A, C, and carotenoids was significantly associated with reduced glaucoma risk. These findings collectively underscore the crucial role of dietary nutrients in glaucoma prevention.

Lifestyle factors have also been extensively investigated in relation to glaucoma. Tseng et al. (29) observed an inverse association between exercise intensity and glaucoma probability. In a cohort study, Pérez-de-Arcelus et al. (30) established a significant correlation between smoking and increased risk of glaucoma development. Marshall et al. (31) reported a positive association between body mass index and intraocular pressure (*β* 0.11/SD; 95% CI [0.06, 0.15]; *p* < 0.001). Additionally, a cross-sectional study from a Korean population survey indicated a correlation between alcohol consumption and elevated intraocular pressure risk (32). These observations emphasize the significance of maintaining a healthy lifestyle in glaucoma prevention.

Our findings demonstrate a significant negative correlation between OBS and glaucoma incidence, suggesting that OBS may serve as a protective factor against glaucoma. While these results align with previous studies, our approach is distinctive in utilizing OBS as an exposure variable. This comprehensive measure not only encompasses multiple dimensions of health factors, thereby overcoming the limitations of single-indicator analyses, but also provides a more accurate reflection of the body’s overall oxidative-antioxidant balance status.

Our subgroup analysis revealed that the protective effects of OBS were more pronounced in middle-aged individuals and males. These age- and gender-specific variations in OBS effectiveness warrant further discussion. With advancing age, there is a documented decline in mitochondrial DNA integrity, reduced oxidative capacity, and increased reactive oxygen species generation. Additionally, age-related tissues exhibit enhanced cellular apoptosis due to mitochondrial dysfunction (33). The diminished protective effect of OBS observed in elderly populations might be attributed to this age-related deterioration in mitochondrial function and weakened antioxidant defense mechanisms. Interestingly, the observed gender differences in OBS effectiveness may be linked to variations in estrogen levels. In a pivotal study, Chen et al. (34) investigated the regulatory role of estrogen in intraocular pressure (IOP) by comparing wild-type mice with those lacking aromatase, an enzyme essential for estrogen production. Their findings demonstrated significantly elevated IOP levels in female aromatase-knockout mice compared to age-matched wild-type controls, while male mice showed no significant differences between groups. Moreover, estrogen deficiency has been implicated in accelerating optic nerve aging processes (35). Clinical evidence further supports this relationship, as studies have shown that prolonged estrogen exposure reduces the risk of primary open-angle glaucoma (POAG) (36), whereas decreased estrogen exposure duration is associated with an increased risk of POAG development (37). Consequently, we hypothesize that the presence of estrogen might partially counteract the protective effects of OBS in females.

This study is the first to demonstrate the association between oxidative balance score and glaucoma risk in a large population and exhibits several notable strengths. First, it is based on the NHANES database with a stratified multistage sampling design, ensuring sample representativeness and result generalizability. Second, we employed a comprehensive oxidative balance scoring system, which provided a more accurate measurement of oxidative stress status. Regarding the analytical strategy, we utilized multivariable logistic regression models, adequately adjusting for potential confounders including demographic characteristics, socioeconomic factors, and comorbidities. Through restricted cubic spline analysis, we revealed a non-linear relationship between OBS and glaucoma risk. Furthermore, our detailed stratification analyses identified significant effect modifications by age and gender, providing important evidence for individualized prevention strategies in clinical practice.

However, this study has several limitations. First, the cross-sectional design based on NHANES database only reveals the association between OBS and glaucoma, preventing causal inference. Second, although we adjusted for multiple potential confounders, unmeasured confounding factors such as genetic factors and medication use might still exist. Third, the study only included US populations, warranting caution when generalizing results to other ethnic groups or populations. Finally, dietary data collected through 24-h recall may not fully reflect participants’ long-term dietary patterns, and the use of dietary supplements was not considered, which may limit the comprehensiveness of the OBS. Therefore, more longitudinal and multicenter studies are needed in the future to validate our findings and further explore the causal relationship between OBS and glaucoma.

## Conclusion

This study investigates the relationship between the OBS and glaucoma risk using NHANES 2005–2008 data. OBS, serving as a comprehensive indicator integrating both antioxidant and pro-oxidant exposures, demonstrated a significant inverse correlation with glaucoma prevalence, suggesting a potential protective effect. Notable demographic variations were observed, with stronger protective associations evident among middle-aged adults and males. These findings provide novel insights into glaucoma prevention and management strategies. However, longitudinal studies are warranted to validate these observations and elucidate the underlying mechanisms.

## Data Availability

Publicly available datasets were analyzed in this study. This data can be found at: https://www.cdc.gov/nchs/nhanes/.
